# The Potential of Using Screening Tools for Bipolar Disorder to Predict Lithium Response

**DOI:** 10.3390/ph18020269

**Published:** 2025-02-18

**Authors:** Delfina Janiri, Mario Pinto, Silvia Montanari, Ester Maria Marzo, Greta Sfratta, Guglielmo Donofrio, Alexia Koukopoulos, Giovanni Camardese, Alessio Simonetti, Lorenzo Moccia, Gianna Autullo, Gabriele Sani

**Affiliations:** 1 Department of Neuroscience, Section of Psychiatry, Università Cattolica del Sacro Cuore, Largo Francesco Vito 1, 00168 Rome, Italy; delfina.janiri@unicatt.it (D.J.); silvia.montanari@unicatt.it (S.M.); estermarzo7@gmail.com (E.M.M.); sfrattagreta24@gmail.com (G.S.); guglielmodonofrio96@gmail.com (G.D.); lorenzo.moccia@unicatt.it (L.M.); 2 Department of Psychiatry, Fondazione Policlinico Universitario Agostino Gemelli IRCCS, Largo Agostino Gemelli 8, 00168 Rome, Italy; mario.pinto@guest.policlinicogemelli.it (M.P.); g.camardese@unilink.it (G.C.); alessio.simonetti@policlinicogemelli.it (A.S.); gianna.autullo@unicatt.it (G.A.); 3 Centro Lucio Bini, ARETÆUS Onlus, Via Crescenzio 42, 00193 Rome, Italy; alexiakoukopoulos@gmail.com; 4 Department of Life Science, Health, and Health Professions, Link Campus University, 00165 Rome, Italy; 5 Menninger Department of Psychiatry and Behavioral Sciences, Baylor College of Medicine, Houston, TX 77030, USA

**Keywords:** lithium, screening, bipolar disorder, hypomania, mania, treatment response, Alda scale

## Abstract

**Background/Objectives:** Lithium is the gold standard for treating Bipolar Disorder (BD), but its effectiveness varies widely. While clinical and environmental factors may influence response, it remains unclear if screening tools can reliably predict lithium response outcomes. This study explores this potential using two widely used screening instruments for BD. **Methods:** A total of 146 patients with BD were evaluated. Lithium response was assessed using the Alda Scale, while hypomanic and manic symptoms were characterized through the Hypomania Checklist-32 (HCL-32) and the Mood Disorder Questionnaire (MDQ). Group differences in HCL-32 and MDQ scores were analyzed using ANOVA, and a multivariate model was employed to identify predictors of lithium response. **Results:** Of the total sample, 46 (31.5%) patients were identified as lithium responders based on the Alda Scale. Responders exhibited significantly higher HCL-32 scores compared to non-responders (*p* = 0.023), while no differences were observed in MDQ scores or other sociodemographic characteristics. Linear regression analysis revealed that HCL-32 scores were a significant predictor of Alda Scale scores, with no associations found for age, gender, or MDQ scores. **Conclusions:** Our study underscores the importance of considering hypomanic symptoms when estimating lithium response in BD, particularly by utilizing the HCL-32 during screening.

## 1. Introduction

Lithium remains the gold standard for treating Bipolar Disorder (BD), yet its effectiveness varies significantly across patients. Approximately 30% of individuals treated with lithium are classified as “excellent responders”, while others show a range of reduced responses [1,2]. The most widely adopted and validated method to assess lithium response is the Retrospective Criteria of Long-Term Treatment Response in Research Subjects with Bipolar Disorder, commonly referred to as the Alda Scale, named after its creator, Martin Alda [3]. The variability in lithium response, even when accounting for potential issues with treatment adherence, highlights the urgent need for reliable predictors of response to personalize treatment strategies and improve clinical outcomes.

Patients who respond well to lithium often display distinct phenotypic traits. These include specific clinical features, such as a mania–depression–interval cycle without rapid cycling, a family history of BD—especially among relatives who also responded well to lithium—and a shorter illness duration prior to starting lithium treatment [4]. Other significant factors, such as the number of hospitalizations before treatment, have also been identified [5,6]. Notably, their response to treatment tends to remain stable over time. Recent studies employing machine learning methods have partially replicated these clinical findings, highlighting additional key variables such as age at first diagnosis, age at first treatment, and the interval between these events as predictors of lithium responsiveness [7,8]. Furthermore, temperament styles and environmental factors, such as childhood abuse, have been shown to influence lithium treatment outcomes [9,10]. Hyperthymic and irritable temperaments are associated with better responses, whereas histories of physical or emotional abuse negatively affect outcomes [10]. These factors likely influence both the pathophysiology of BD and adherence to treatment, ultimately shaping its effectiveness [11].

While clinical predictors of lithium response have been extensively studied, it remains unclear whether screening tools for BD can serve as quantitative instruments to predict treatment outcomes. Two of the most widely used tools for the early identification and characterization of mood disorders, including BD, are the Mood Disorder Questionnaire (MDQ; [12]) and the Hypomania Checklist-32 (HCL-32; [13]). These tools are well-established for detecting BD and demonstrate strong overall diagnostic accuracy [14,15]. However, no definitive evidence has yet linked the results of these instruments to lithium response.

Given the variability in lithium response, tools and guidelines that improve the prediction of positive outcomes are invaluable for clinicians and patients [16]. This study aimed to investigate whether the MDQ and HCL-32, the primary screening instruments for BD, could also serve as predictors of lithium response.

## 2. Results

In the total sample, 76 participants were male (52%), and 70 were female (48%). The mean age was 43.78 years (SD = 12.33), and the mean education level was 14.38 years (SD = 4.20). The classification of participants in our study, based on their response to lithium treatment according to the Alda Scale criteria, identified 46 (31.5%) patients as treatment Responders (mean TS = 7.46, SD = 0.66) and 100 (68.5%) patients as Non-Responders (mean TS = 2.92, SD = 1.76). Sociodemographic and clinical characteristics of the sample are shown in Table 1.

**Figure 1:**
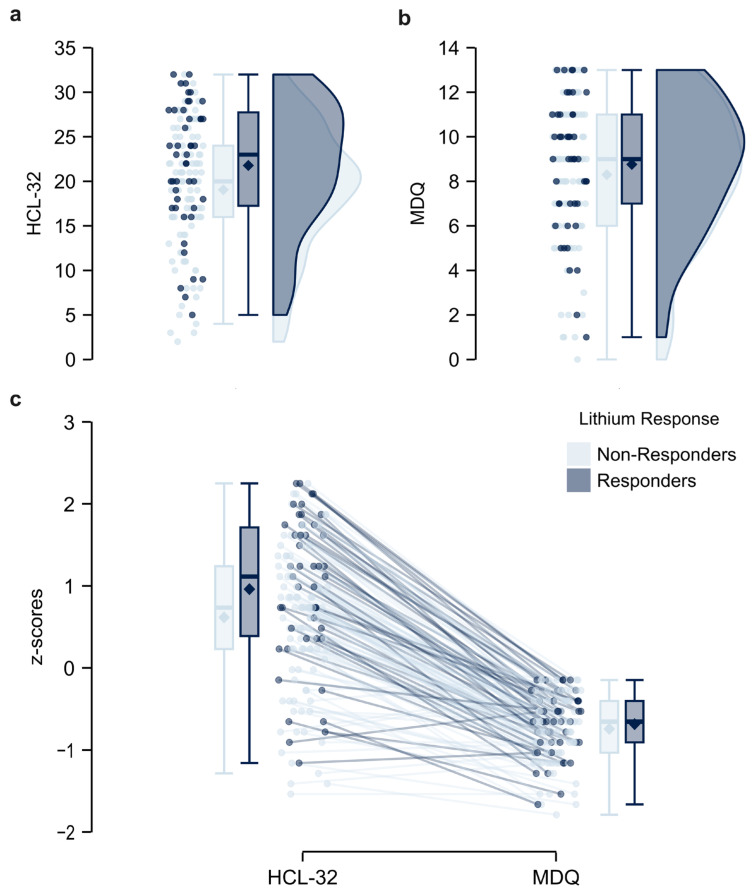
Raincloud plots illustrating the distribution of scores obtained by Lithium Non-Responders and Lithium Responders on (**a**) the Hypomania Checklist-32 (HCL-32) and (**b**) the Mood Disorder Questionnaire (MDQ). (**c**) Distributions of aggregate z-scores, computed across the HCL-32 and MDQ, are shown with corresponding box plots as a function of group classification into Lithium Non-Responders and Lithium Responders.

The two groups of patients did not differ in their sociodemographic and clinical characteristics, except for the alternative treatments, which were higher for the Lithium Non-Responders group (see Table 1). Regarding the screening assessment, the two groups of patients presented with a significant difference in the HCL-32 scores [F(1, 144) = 4.84, *p* = 0.023, η^2^ₚ = 0.051; Levene’s Test for Equality of Variances: *p* = 0.600], highlighting that patients classified as Lithium Responders had higher scores on the HCL-32 compared to patients classified as Lithium Non-Responders (Figure 1a). Conversely, the results showed no difference in the scores of MDQ between the two group of patients [F(1, 44) = 0.70, *p* = 0.403, η^2^ₚ = 0.005; Levene’s Test for Equality of Variances: *p* = 0.765] (Figure 1b).

**Figure 2:**
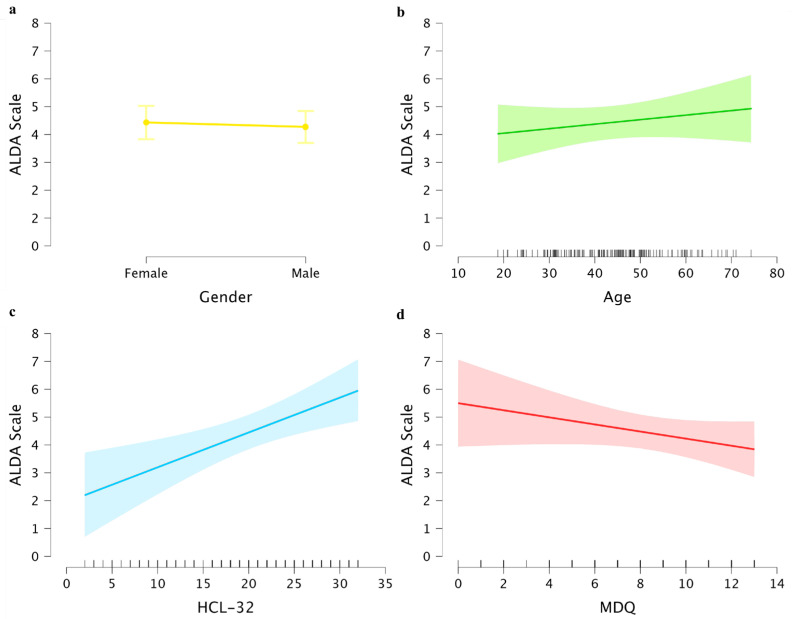
Marginal effects of the linear regression analysis. The plot illustrates the relationships between the predictor variables (**a**) Gender, (**b**) Age, (**c**) HCL-32, and (**d**) MDQ and the Alda Scale scores, highlighting the estimated regression lines along with their confidence intervals.

The linear regression analysis conducted on the Alda Scale total scores revealed a significant regression model [F(4, 145) = 2.87, *p* = 0.023], which explained a significant portion of the variance in the scores obtained (R^2^ = 0.074, Adjusted R^2^ = 0.056). In detail, only the HCL-32 scores were found to be a significant direct predictor (β = 0.342, t = 3.260, *p* = 0.001; see Figure 2c), showing that higher scores obtained in the HCL-32 predicted higher scores obtained in the Alda Scale. By contrast, no significant association was found with gender (β = −0.159, t = −0.381, *p* = 0.704, see Figure 2a), age (β = 0.077, t = 0.925, *p* = 0.356; see Figure 2b) or MDQ scores (β = −0.151, t = −1.466, *p* = 0.145; see Figure 2d).

In our regression analyses, The Mahalanobis Distance was smaller than the critical value (all *p*  >  0.001, critical value recommended by Tabachnick et al. [17]) indicating that in our set of data, no multivariate outliers were present. In addition, we found that the distribution of variables was comparable to a multivariate normal distribution (Mardia’s multivariate kurtosis index  =  15.21; reference value  =  24; [18,19]).

Regarding multicollinearity, the obtained values in this study (Age: Tolerance = 0.953, VIF = 1.050; Gender: Tolerance = 0.999, VIF = 1.001; HCL: Tolerance = 0.595, VIF = 1.680; MDQ: Tolerance = 0.616, VIF = 1.624) were all above 0.10 for Tolerance, indicating low multicollinearity, and the VIF values for the independent variables were below 4, suggesting no significant collinearity issues.

## 3. Discussion

The aim of our study was to assess whether clinical tools commonly used for psychopathological screening in the diagnosis of bipolar disorder, specifically the Mood Disorder Questionnaire (MDQ) and the Hypomania Checklist-32 (HCL-32), could also serve as predictive variables for a positive response to lithium treatment.

Our findings revealed that the Lithium Responder group scored significantly higher on the HCL-32 compared to the Non-Responder group, while no significant differences were observed in MDQ scores between the two groups. Furthermore, regression analysis demonstrated a significant predictive relationship between the HCL-32 and lithium response. Specifically, patients with more hypomanic symptoms achieved higher scores on the Alda Scale, indicating a strong association between HCL-32 scores and lithium responsiveness (*p* = 0.023).

These findings align with previous research emphasizing the link between manic/hypomanic features and improved lithium response. Earlier studies have demonstrated that patients with a manic–depressive interval (MDI) type of cycle are more likely to respond favorably to lithium treatment [4,9,10]. In parallel, patients with an MDI cycle tend to exhibit more pronounced manic/hypomanic symptoms compared to patients with a depressive–manic interval (DMI) cycle, who generally experience less manic morbidity during long-term follow-up but show reduced responsiveness to lithium [20,21]. Our results also align with lithium’s well-established antimanic effects. Lithium is particularly effective when initiated during a manic or hypomanic phase, serving as a protective factor against subsequent depressive episodes in individuals with MDI cycles [20,22]. A recent study conducted by our group also found that a hyperthymic temperament predicts a favorable response to lithium treatment [10], supporting previous research that identified a positive correlation between lithium response and hyperthymic temperament [23]. Notably, hyperthymic traits are often linked to a predominant manic/hypomanic polarity [24,25,26,27], which, in turn, predicts a positive lithium response phenotype [25]. These results are consistent with the long-standing view of lithium’s primary antimanic effect, first observed by Cade [28], and align with the association we found in the current study between higher scores on the Alda Scale and the HCL-32. Moreover, previous research suggests that lithium may influence gene expression, acting as a neurotrophic factor [29,30,31]. Interestingly, other studies have reported a link between genes associated with manic predominant polarity and hyperthymic temperament [32,33,34,35]. Future investigations should therefore further explore the promising yet still preliminary results obtained so far from genetic studies on lithium response in bipolar patients. [36,37,38].

The strong association between HCL-32 scores and lithium responsiveness may be attributed to the tool’s sensitivity to manic and hypomanic polarity. These components of bipolar disorder—characterized by impulsivity, overactivity, elevated mood, and increased energy—are effectively captured by the HCL-32 [13,39], which has demonstrated robust diagnostic sensitivity. In contrast, our study found no predictive relationship between MDQ scores and lithium responsiveness. This discrepancy may be explained by the broader scope of MDQ items, some of which can reflect conditions beyond bipolar disorder, such as anxiety, trauma-related disorders, substance use disorders, eating disorders, and impulse control disorders [40,41]. Previous studies have highlighted this limitation by identifying a two-factor structure within the MDQ, consisting of Positive Activation and Negative Activation dimensions [42,43]. The Positive Activation subscale—which includes symptoms such as increased energy, grandiosity, and decreased need for sleep—is specific to bipolar disorder. In contrast, the Negative Activation subscale—encompassing symptoms like irritability, racing thoughts, negative emotional states, and distractibility—is more broadly associated with emotion dysregulation and transdiagnostic personality traits [42,43]. While the MDQ remains a valuable screening tool for bipolar disorder, its predictive value for lithium response may be limited by its reduced specificity for manic polarity.

Our study, along with the existing literature, highlights the distinct qualities of the HCL-32 and MDQ in predicting lithium response. The HCL-32, with its sensitivity to manic traits, is better for identifying patients likely to respond to lithium. In contrast, the MDQ’s broader scope makes it a useful screening tool, though less specific for lithium response. These findings emphasize the importance of personalized treatment, suggesting that detailed clinical phenotypes may offer more insight into lithium response than traditional diagnostic categories [44] and could lead to more tailored and effective pharmacological strategies.

Before presenting the conclusions, we must acknowledge certain limitations that may affect the generalizability of our findings. First, the cross-sectional design of our study limits our ability to establish causal relationships. However, we mitigated this limitation by using a multivariate model to predict lithium response. Second, clinical variables and treatment response were assessed retrospectively. Nevertheless, we employed the Alda Scale, which is considered the gold standard for evaluating lithium response [45]. Furthermore, to reduce the risk of recall bias, we gathered information not only from patient reports but also from family members and close friends (who were present at least at one visit) and reviewed all available medical records. Additionally, the study population was limited to outpatients from a single clinical site, so the results may not be widely applicable. Nonetheless, this study represents the first attempt to consider screening tools for BD as predictors of lithium response.

The results of our study may have important therapeutic implications in the future. The significant predictive value of the Hypomania Checklist-32 for lithium treatment response could enable more personalized treatment plans. Future studies could expand on these findings, focusing on improving the use of psychometric tools for predicting lithium response, particularly emphasizing the importance of manic polarity in treatment stratification. Additionally, future research could explore the integration of psychopathological tools with biological markers, such as genetic biomarkers. These findings support the growing effort to optimize treatment approaches for bipolar disorder through accurate phenotypic and psychometric assessments.

## 4. Materials and Methods

### 4.1. Participants

We enrolled 146 outpatients with DSM-5 diagnoses of BD type I and BD type II. Patients were recruited from the Psychiatry Department of the Fondazione Policlinico Universitario Agostino Gemelli IRCCS in Rome, Italy. Diagnosis was confirmed using the Structured Clinical Interview for DSM-5 [46]. In addition to a DSM-5 diagnosis of BD, inclusion criteria were as follows: (a) age 18–65 years; (b) at least 5 years of education; (c) fluency in Italian; (d) stable pharmacological treatment with lithium for at least 3 months. Exclusion criteria were (a) a history of psychosis not related to the primary mood disorder; (b) traumatic brain injury with loss of consciousness; (c) major medical or neurological conditions; (d) a Mini-Mental State Examination (MMSE) score of less than 24 (since scores below this level indicate cognitive deterioration based on normative data from the Italian population); (e) current substance use disorder. Patients were in clinical remission according to the Young Mania Rating Scale (YMRS) for manic symptoms and the 17-item Hamilton Rating Scale for Depression (HAMD) for depressive symptoms.

The study adhered to the Principles of Human Rights established by the World Medical Association (WMA) at the 18th General Assembly in Helsinki, Finland (June 1964), and subsequently revised at the 64th General Assembly in Fortaleza, Brazil (October 2013). All participants provided written informed consent after receiving a comprehensive explanation of the objectives and procedures of the study. No financial compensation was offered to participants. The research followed strict ethical standards, ensuring full protection of participants’ rights and well-being. This adherence to ethical principles, combined with thorough diagnostic procedures, reinforces both the scientific rigor and ethical integrity of the study, enhancing the validity and reliability of its findings. The study received approval from local ethics committees (protocol number: 5016, 23 January 2023).

### 4.2. Clinical and Psychopathological Assessment

A semi-structured interview, used in prior studies [47], was employed to collect comprehensive data on anamnestic characteristics and clinical information. Administered by an experienced psychiatrist, the interview adhered to DSM-5 criteria and clinical assessments, avoiding simplistic yes/no responses to ensure more nuanced insights. The wording of questions was flexible for clarity, and the final evaluation incorporated input not only from the patients but also from family members or close friends (who were present for at least one visit) and relevant medical records.

To characterize bipolar disorders [15], two additional tools were administered: the Hypomania Symptom Checklist-32 (HCL-32) and the Mood Disorder Questionnaire (MDQ).

The HCL-32 is a self-report questionnaire commonly used to detect hypomanic symptoms, particularly in individuals with bipolar spectrum disorders [13,39]. It consists of 32 items that evaluate behaviors and experiences associated with elevated mood, increased energy, overactivity, and impulsivity. Participants respond in a dichotomous format (yes/no) based on whether they have experienced specific symptoms during their “highs”. A total score is calculated by summing the “yes” responses, with higher scores suggesting a greater likelihood of bipolarity. The commonly used cut-off is ≥14, though thresholds may vary depending on the clinical or research context.

The MDQ is a 15-item self-report screening instrument designed to identify individuals most likely to have bipolar disorder [12,48]. It includes 13 yes/no questions about manic or hypomanic symptoms, a question on symptom co-occurrence, and one assessing symptom-related functional impairment. It is particularly useful in distinguishing bipolar disorder from other mood disturbances. A positive screening result typically requires a “yes” to at least 7 out of 13 symptoms, symptom co-occurrence, and moderate or severe functional impairment.

### 4.3. Lithium Response Assessment

To assess the response to lithium treatment, we employed the Alda Scale, which is specifically designed for the retrospective evaluation of prophylactic treatment responses in naturalistic settings. The instrument consists of two subscales, labeled A and B. The A scale, which ranges from 0 to 10, measures the degree of improvement attributed to lithium treatment, including reductions in the frequency of recurrences and residual symptoms, among other factors. The B scale contains five criteria, each rated from 0 to 2, with higher scores indicating a greater likelihood that external factors may have influenced the observed improvements. The five potential confounding factors in the B scale are as follows: (B1) number of episodes before or off treatment, (B2) frequency of episodes before or off treatment, (B3) duration of lithium treatment, (B4) compliance during periods of stability, and (B5) use of additional medication during periods of stability. The total score (TS) on the Alda Scale is calculated by subtracting the B score from the A score. If the B score exceeds the A score, resulting in a negative value, it is recorded as 0.

In accordance with Manchia et al. [45], a TS value of 7 or higher was considered indicative of a respondent phenotype. Although the Alda scale was originally designed to evaluate long-term treatment responses, we chose to include participants with less than one year of lithium treatment in our study. This decision was made because the scale accounts for potential bias due to treatment duration under Criterion B3. It is important to note that only a small proportion (2.5%) of our sample had been receiving lithium treatment for less than a year.

### 4.4. Statistical Analyses

First, we divided our sample into two groups: Lithium Responders and Lithium Non-Responders, based on the Alda Scale cut-off (TS ≥ 7 indicating good lithium responders).

Second, we conducted a series of Chi-Squared Tests and one-way ANOVAs with lithium response as the independent variable and sociodemographic and clinical variables and the scores obtained on the screening assessment (HCL-32 or MDQ) as the dependent variables. The aim was to evaluate potential differences in these variables between patient groups classified as Responders or Non-Responders to lithium treatment. Significance was set at *p* = 0.05. To account for multiple comparisons and reduce the likelihood of Type I errors, a Bonferroni correction was applied to the results (adjusted *p*-value: *p* < 0.05/number of comparisons).

Third, we conducted a linear regression analysis to assess the contribution of HCL-32 and MDQ scores as predictor variables for lithium response. The TS obtained on the Alda Scale was used as the dependent variable, while the HCL-32 and MDQ scores, together with age and gender, were included as independent variables. This approach allowed us to explore the influence of each predictor on the responses of patients to lithium treatment, as assessed by the Alda Scale. Multivariate normality in the regression analyses was regularly assessed [49]. Multicollinearity between the predictor variables was assessed using Tolerance and VIF values. All statistical analyses were performed using JASP (Version 0.19.1; JASP Team, 2024) and SPSS (Version 29.0.1.0; IBM Corp, Armonk, NY, USA, 2023).

## Figures and Tables

**Table 1:** Sociodemographic, clinical, and screening assessment of the sample according to lithium response (N = 146).

Variables	Lithium Responders (N = 46)	Lithium Non-Responders (N = 100)	F or χ^2^	df	*p* Value	Effect Size (η^2^ₚ or φ)
Age, y—mean ± SD	43.60 ± 13.09	43.86 ± 11.90	0.01	1	0.907	9.46 × 10^−5^
Gender, N (%)			<0.01	1	0.987	0.002
Male	24 (52.2%)	52 (52.0%)				
Female	22 (47.8%)	48 (48.0%)				
Education, y—mean ± SD	14.67 ± 3.41	14.25 ± 3.49	0.47	1	0.494	0.003
Married, yes—N (%)	19 (41.3%)	45 (45.0%)	0.18	1	0.676	0.035
Coffee, yes—N (%)	36 (78.3%)	78 (78.0%)	< 0.01	1	0.972	−0.003
Smoking, yes—N (%)	23 (50.0%)	48 (48.0%)	0.05	1	0.822	0.019
Family psychiatric history, yes—N (%)	33 (71.8%)	68 (68.0%)	0.21	1	0.649	0.038
Hospitalization, yes—N (%)	27 (58.7%)	70 (70.0%)	1.81	1	0.179	0.111
Body Mass Index, mean ± SD	25.67 ± 4.35	25.84 ± 3.68	0.06	1	0.813	3.90 × 10^−4^
Hypomania Symptom Checklist-32 (HCL-32), mean ± SD	21.78 ± 7.17	19.05 ± 6.92	4.80	1	0.023 *	0.051
Mood Disorder Questionnaire (MDQ), mean ± SD	8.76 ± 2.99	8.30 ± 3.12	0.70	1	0.403	0.005
Antidepressants, yes—N (%)	7 (15.2%)	38 (38.0%)	7.760	1	0.006 *	0.223
Antipsychotics, yes—N (%)	26 (56.5%)	78 (78.0%)	7.093	1	0.008 *	0.215
Antiepileptics, yes—N (%)	18 (39.1%)	59 (59.0%)	4.991	1	0.025 *	0.182

* *p* < 0.05; N, number of observations; df, degrees of freedom; y, years; SD, standard deviation.

## Data Availability

The data presented in this study are available on request from the corresponding author due to ethical reasons.
